# Development of a Phosphoric Acid-Mediated Hyaluronic Acid Gel Sheet for Efficient Transdermal Delivery of Alendronate for Anti-Osteoporotic Therapy

**DOI:** 10.3390/pharmaceutics11120643

**Published:** 2019-12-02

**Authors:** Chihiro Naito, Hidemasa Katsumi, Kunio Yoneto, Mao Omura, Mayuko Nishidono, Sachi Kamei, Akiya Mizoguchi, Ayaka Tamba, Akiko Tanaka, Masaki Morishita, Akira Yamamoto

**Affiliations:** 1 Department of Biopharmaceutics, Kyoto Pharmaceutical University, Yamashina-ku, Kyoto 607-8414, Japan; kd16008@poppy.kyoto-phu.ac.jp (C.N.); kyoneto04@gmail.com (K.Y.); ky16061@ms.kyoto-phu.ac.jp (M.O.); ky16256@ms.kyoto-phu.ac.jp (M.N.); ky15099@ms.kyoto-phu.ac.jp (S.K.); ky15328@ms.kyoto-phu.ac.jp (A.M.); kurukuru1.5@outlook.jp (A.T.); a-tanaka@kobepharma-u.ac.jp (A.T.); morishita@mb.kyoto-phu.ac.jp (M.M.); yamamoto@mb.kyoto-phu.ac.jp (A.Y.); 2 Ritapharma, Co., Ltd., Shimogyo-ku, Japan, Kyoto 600-8813, Japan

**Keywords:** hyaluronic acid, gel sheet, alendronate, transdermal absorption, osteoporosis

## Abstract

For efficient transdermal delivery of alendronate (ALN) for anti-osteoporotic therapy, we developed a hyaluronic acid (HA) gel sheet that was prepared simply by enhancing HA noncovalent interactions using phosphoric acid and polyhydric alcohol (propanediol and glycerin). HA solution viscosity increased after addition of phosphoric acid, and the HA gel sheet formed after heated drying. The HA gel sheet could be converted to high viscosity state by addition of water. These results indicate that phosphoric acid enhances the noncovalent interactions of HA molecules. The HA gel sheet elicited no skin irritation over 7 days after a 24-h application. The permeation of ALN across rat and human skin was 109 and 7.17 µg/cm^2^, respectively, up to 24 h after application of the ALN-loaded HA gel sheet, which is sufficient for clinical treatment of osteoporosis. The bioavailability of ALN in rats was ~20% after application of the ALN-loaded HA gel sheet, and plasma calcium levels were effectively reduced 3 days after sheet application. Furthermore, in a rat osteoporosis model, the reduction in tibial bone density was suppressed by treatment with the ALN-loaded HA gel sheet. These results indicate that our phosphoric acid-mediated HA gel sheet is a promising transdermal formulation for efficient ALN delivery.

## 1. Introduction

Alendronate (ALN) is a bisphosphonate that inhibits osteoclastic bone resorption and is widely used as a first-line treatment for postmenopausal osteoporosis [1]. It was reported that ALN increased bone mineral density in women with postmenopausal osteoporosis [2] and decreased the risk of vertebral and nonvertebral fractures, including hip fractures [3]. However, the intestinal absorption of ALN after oral administration is limited due to its high polarity and hydrophilicity [4]. Furthermore, ALN can cause serious gastrointestinal side effects, such as diarrhea, abdominal pain, and inflammation, and erosions and ulceration of the upper gastrointestinal tract [5]. To prevent these side effects, patients should sit up for at least 30 min after the oral administration of ALN, which can be difficult for some elderly patients. Therefore, the development of alternative formulations is required to improve adherence and the quality of life of patients who are being treated with ALN and to improve its therapeutic efficacy.

Of the various strategies available, transdermal delivery is considered an attractive administration method for ALN because it is painless and easy to apply in both elderly and bedridden patients. In addition, transdermal delivery can avoid side effects, including intestinal damage, caused by ALN. Recently, several groups have reported transdermal ALN delivery systems. Choi et al. [6,7] and Boche et al. [8] developed transdermal formulations of ALN. Furthermore, we reported the efficient transdermal delivery of ALN using a hydrophilic patch system [9]. We also used hyaluronic acid (HA) as a base material for the microneedle arrays for transdermal ALN delivery [10,11], because HA possesses versatile properties such as biocompatibility, non-immunogenicity, biodegradability, and viscoelasticity [12]. However, these technologies have not been applied clinically to date due to the complexities in manufacturing these technologies and devices.

HA gel sheets are widely used for medical treatment such as wound healing and bone regeneration [13,14]. They are usually prepared by crosslinking HA via chemical modification [15,16,17,18,19,20], but this modified HA is not natural and its manufacturing process is complicated. For the efficient transdermal delivery of ALN, the aim of this study was to develop an HA gel sheet that is produced simply by noncovalent interactions. To this end, we designed a phosphoric acid-mediated, HA-based gel sheet that is simply prepared by the physical mixing of HA, phosphoric acid, and polyhydric alcohol (propanediol and glycerin). Using ALN-loaded HA gel sheets, we then investigated the skin permeation and absorption of ALN using rat and human skin, and the therapeutic potential of ALN after the transdermal application of the sheets in a rat model.

## 2. Materials and Methods

### 2.1. Materials

Alendronate sodium trihydrate was obtained from Toronto Research Chemicals, Inc. (North York, ON, Canada). [^14^C]ALN sodium salt was purchased from Moravek, Inc. (Brea, CA, USA). HA (HYALURONSAN HA-LQSH, average molecular weight; 2300 kDa) was obtained from Kewpie Corporation (Tokyo, Japan). Propanediol, glycerin, and phosphoric acid were purchased from FUJIFILM Wako Pure Chemicals Industries, Ltd. (Osaka, Japan). Human cadaver skin, for which full ethical committee approval and informed patient consent were obtained, was purchased from Platinum TRAINING (Henderson, NV, USA). This study was approved by the ethical committee on human research of Kyoto Pharmaceutical University. All other chemicals were obtained commercially as reagent-grade products.

### 2.2. Animals

Male Wistar rats (250–270 g) and male and female Sprague Dawley rats (180–200 g) were purchased from Japan SLC, Inc. (Shizuoka, Japan). All animal experiments were conducted in accordance with the principles and procedures outlined in the National Institutes of Health Guidelines for the Care and Use of Laboratory Animals. The protocols for animal experiments were approved by the Animal Experimentation Committee of Kyoto Pharmaceutical University (Permit number 18-17-027) (Date of approval: 26 April 2018).

### 2.3. Preparation of ALN-Loaded HA Gel Sheets

HA (100 mg) was dissolved in 10 g of distilled water and heated at 60 °C until the HA was hydrated. The HA solution was mixed with glycerin (0.8 g) and propanediol (3.2 g). Four grams of the obtained HA–polyhydric alcohol solution was added to each well of a 6-well plate. Phosphoric acid (1420 μL of a 1% solution) and 1164 µL of ALN solution (5 mg/mL) were added to the HA–polyhydric alcohol solution and the mixture was dried for 2 days at 50 °C to obtain the ALN-loaded HA gel sheets. The theoretical concentrations of HA, propanediol, glycerin, phosphoric acid, and ALN in the final dried product (ALN-loaded HA gel sheets) were 2.4%, 76.7%, 19.2%, 1.2%, and 0.5%, respectively. Blank HA gel sheets without ALN were also prepared using the same method as described above.

### 2.4. Effect of Phosphoric Acid and Water on HA Gelation

HA–polyhydric alcohol solutions with and without phosphoric acid and their dried products were prepared by the same method as described in Section 2.3 above. The samples were transferred into the Teflon cup reservoir of a Brookfield Digital Rotational Viscometer (Brookfield Engineering Labs., Inc., Middleboro, MA, USA). The parameters were acquired at 25 °C within 2 min after the onset of the experiment. The Teflon spindle rotation rate of the viscometer was 1 rpm at a shear rate of 3.84 s^−1^.

### 2.5. Skin Irritation After Application of HA Gel Sheets in Rats

The HA gel sheets were applied to the abdomens of the rats and were removed 24 h later. Skin irritation was assessed by observing the application sites as described previously [21,22].

### 2.6. In Vitro Permeation Study Using Rat and Human Skin

The abdomen of each Wistar rat was shaved using an animal hair clipper under anesthesia using a mixture of medetomidine (0.3 mg/kg), midazolam (2 mg/kg), and butorphanol (2.5 mg/kg). The rats were euthanized and the full thickness of abdominal skin was excised. Excess fat adhering to the dermis side was removed using cotton. Franz diffusion cells were used for the permeation study. A piece of excised skin (human or rat, area of 3.1 cm^2^, diameter of 20 mm) was mounted between the receptor and donor chambers with the stratum corneum facing the donor compartment. An ALN-loaded HA gel sheet (one, 0.785 cm^2^ piece containing 0.5 mg of ALN) was applied to the stratum corneum side of the rat skin. [^14^C]ALN-loaded HA gel sheet (one, 0.95 cm^2^ piece containing 0.625 mg of ALN) was applied to the stratum corneum side of the human skin. The receptor compartment was filled with 3 mL of phosphate-buffered saline (PBS, pH 7.4). The Franz cells were incubated at 32 °C and stirred with magnetic bars. Receptor solution (0.3 mL) was withdrawn periodically and replaced with an equal volume of fresh PBS [9]. The concentration of ALN that had permeated across the rat skin in each sample was analyzed using the reversed-phase high-performance liquid chromatography (RP-HPLC) method reported previously [23]. The [^14^C]ALN that had permeated across the human skin in each sample (100 μL) was mixed with 5 mL Clear-Sol I (Nacalai Tesque, Inc., Kyoto, Japan). The samples were stored overnight, and the radioactivity was then measured using a scintillation counter (LSC-6100, Aloka, Tokyo, Japan).

### 2.7. Pharmacokinetics of ALN in Rats

Under anesthesia, using a mixture of medetomidine (0.3 mg/kg), midazolam (2 mg/kg), and butorphanol (2.5 mg/kg), [^14^C]ALN-loaded HA gel sheet was applied on the shaved abdomen of each Wistar rat at a dose of 2.5 mg ALN/kg. [^14^C]ALN was also intravenously administered in the femoral vein of separate rats at a dose of 1 mg/kg. At predetermined intervals, blood was collected from the jugular vein. The resulting plasma (100 μL) was oxidized with 100 μL of 30% hydrogen peroxide. Then, 5 mL Clear-Sol I was added to each sample. The samples were stored overnight, and the radioactivity was measured using a scintillation counter (LSC-6100, Aloka, Tokyo, Japan). The concentrations of [^14^C] radioactivity in the plasma after intravenous injection were normalized with respect to the percentage of dose/ml, which was then converted to ng/ml, and analyzed using the nonlinear least-squares program MULTI [24]. The area under the concentration–time curve (AUC) after intravenous injection was calculated based on a two-compartment model. The AUC after transdermal administration was calculated by the trapezoidal rule from time zero to the last time point [25].

### 2.8. Effect of ALN-Loaded HA Gel Sheets on Plasma Calcium Levels

The abdomens of male Sprague Dawley rats were shaved under anesthesia using a mixture of medetomidine (0.3 mg/kg), midazolam (2 mg/kg), and butorphanol (2.5 mg/kg). On day 0, ALN-loaded HA gel sheets were applied to the shaved abdomens for a period of 24 h at a dose of 6.0 mg/rat (three pieces, 3.14 cm^2^ piece of gel sheet containing 2.0 mg of ALN). On days 0 and 3, blood was withdrawn from the jugular veins while the animals were under anesthesia using a mixture of medetomidine (0.3 mg/kg), midazolam (2 mg/kg), and butorphanol (2.5 mg/kg). Plasma was obtained from the blood after centrifugation. Plasma calcium concentrations were measured using Calcium E Test Kit (Fujifilm Wako Pure Chemical Industries, Osaka, Japan) [9].

### 2.9. Therapeutic Potential of ALN-Loaded HA Gel Sheets for Treatment of Osteoporosis

A postmenopausal rat model of osteoporosis was established by performing ovariectomies (OVX) of female Sprague Dawley rats [10,26]. Four weeks after OVX, ALN-loaded HA gel sheets were applied to the shaved abdomens of the OVX rats for a period of 24 h every 2 weeks at a dose of 6.0 mg/rat (three pieces, 3.14 cm^2^ piece of gel sheet containing 2.0 mg of ALN). Separate OVX rats were subcutaneously injected with 1 mg/kg ALN every 2 weeks. Eight weeks after OVX, the rats were euthanized, and their tibias were excised, fixed using 4% paraformaldehyde in PBS, embedded in paraffin, and sectioned by microtome. The tibial sections were stained with hematoxylin and eosin and observed by microscopy (BIOZERO^®^; Keyence, Osaka, Japan).

### 2.10. Statistical Analysis

Results are expressed as means ± standard errors (SE), and statistical significance was assessed by one-way analysis of variance followed by the Student−Newman−Keuls multiple comparison test for multiple groups at a significance level of *p* < 0.05.

## 3. Results

### 3.1. HA Gelation

**Figure 1:**
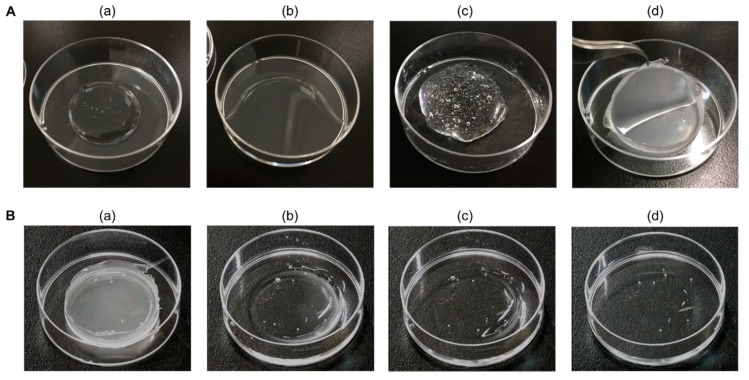
Preparation and reversibility of alendronate (ALN)-loaded hyaluronic acid (HA) gel sheet. (**A**) Preparation of ALN-loaded HA gel sheet. Mixture of (**a**) HA and distilled water; (**b**) HA, distilled water, glycerin, and propanediol; (**c**) HA, distilled water, glycerin, propanediol, ALN, and phosphoric acid before heated drying and (**d**) after heated drying. (**B**) Reversibility process of ALN-loaded HA gel sheet after addition of distilled water. (**a**) Before addition of distilled water, (**b**) 15 min, (**c**) 30 min, and (**d**) 60 min after addition of distilled water.

Figure 1 shows images of the preparation and reversibility of an ALN-loaded HA gel sheet. The viscosity of HA increased after the addition of phosphoric acid. After heated drying, the formation of the HA gel sheet was confirmed (Figure 1A). After addition of distilled water to the sheet, it returned to its high viscosity state (Figure 1B).

### 3.2. Effect of Phosphoric Acid on HA Gelation

To evaluate the effect of phosphoric acid on HA gelation, we measured the viscosity of the HA–polyhydric alcohol solution with and without phosphoric acid (Table 1). The viscosity of the HA–polyhydric alcohol solution with phosphoric acid (approximately 8 Pa·s) was much higher than that of the HA–polyhydric alcohol solution without phosphoric acid (approximately 1.5 Pa·s). After heated drying, the HA–polyhydric alcohol solution without phosphoric acid was still liquid (viscosity; approximately 51 Pa·s), but the HA–polyhydric alcohol solution with phosphoric acid formed a sheet (viscosity; out of range).

### 3.3. Skin Irritation After Application of HA Gel Sheets to Rat Abdominal Skin

**Figure 2:**
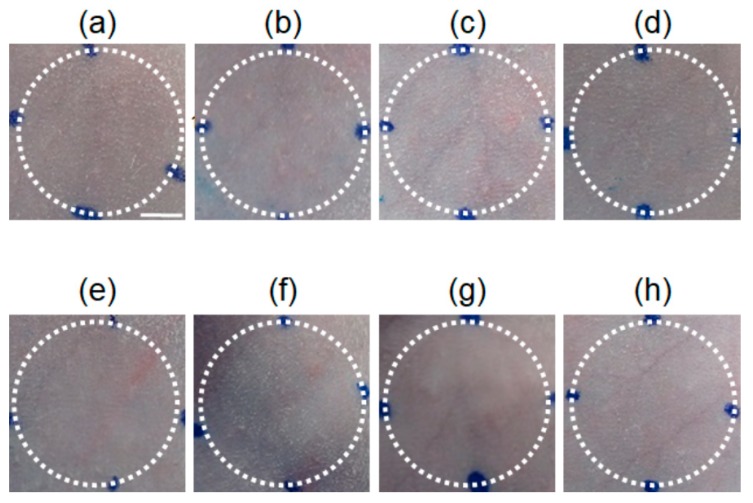
Skin irritation after application of HA gel sheet to rats. Representative images of rat skin (**a**) before application of HA gel sheet, (**b**) 1 day, (**c**) 2 days, (**d**) 3 days, (**e**) 4 days, (**f**) 5 days, (**g**) 6 days, and (**h**) 7 days after application of HA gel sheet. Scale bar represents 5 mm.

To investigate the safety of the HA gel sheets when used on skin, we evaluated localized skin irritation after the 24-h application of HA gel sheets to the abdominal skin of rats (Figure 2). No skin erythema or edema was observed over the 7-day observation period after removal of the HA gel sheet.

### 3.4. Skin Permeation of ALN After Application of ALN-Loaded HA Gel Sheets

**Figure 3:**
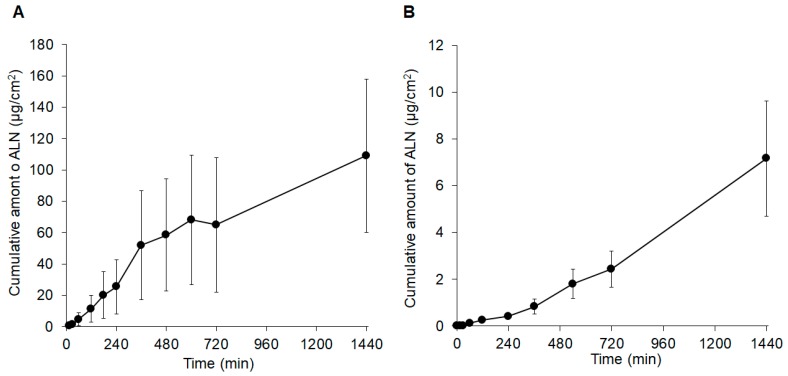
Skin permeation profiles of ALN after application of ALN-loaded HA gel sheets to (**A**) rat skin or (**B**) human skin. Results are expressed as the mean ± standard error (SE) of three experiments.

Figure 3 shows the skin permeation of ALN after application of the ALN-loaded HA gel sheet. The permeation of ALN across rat and human skin was 109 and 7.17 μg/cm^2^, respectively, 24 h (1440 min) after application of the ALN-loaded HA gel sheet.

### 3.5. Transdermal Absorption of ALN After Application of ALN-Loaded HA Gel Sheets

**Figure 4:**
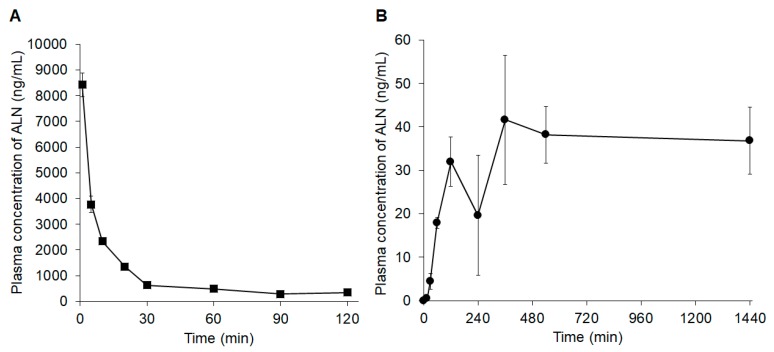
Plasma concentration profiles of ALN (**A**) after intravenous administration of ALN or (**B**) after application of ALN-loaded HA gel sheets. Results are expressed as the mean ± standard error (SE) of three experiments.

Figure 4 and Table 2 show the plasma concentration profiles and pharmacokinetic parameters of ALN in rats after intravenous injection or application of the ALN-loaded HA gel sheets. ALN was rapidly eliminated from the blood circulation by 90 min after intravenous injection (Figure 4A). In contrast, the plasma concentration of ALN gradually increased and reached its maximum 360 min after application of the ALN-loaded HA gel (Figure 4B). The bioavailability of ALN was approximately 20% after application of the ALN-loaded HA gel to rats (Table 2).

### 3.6. Effect of ALN-Loaded HA Gel Sheets on Plasma Calcium Levels in Rats

**Figure 5:**
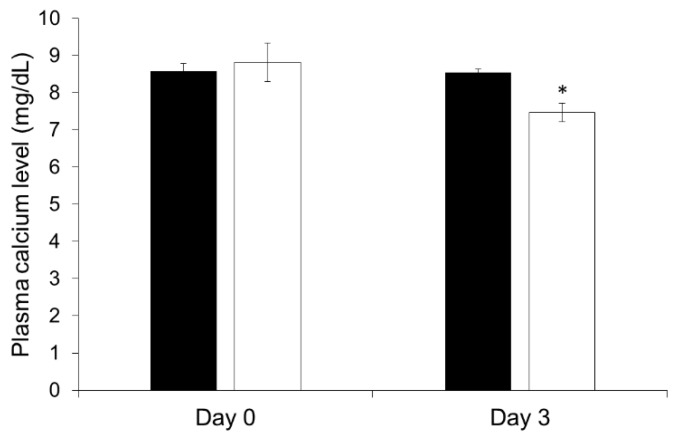
Effect of ALN-loaded HA gel sheet on the plasma calcium level in rats. Black bar, naïve group; white bar, application of ALN-loaded HA gel sheets at 6.0 mg/rat. Results are expressed as the mean ± standard error (SE) of 3–4 experiments. * *p* < 0.05; significant difference compared with plasma calcium level in the naïve group on day 3.

Figure 5 shows the plasma calcium levels of rats after transdermal administration of ALN. No significant change in the plasma calcium levels of naïve rats was observed. In contrast, the plasma calcium levels were reduced 3 days after the application of ALN-loaded HA gel sheets at 6.0 mg/rat.

### 3.7. Therapeutic Potential of ALN-Loaded HA Gel Sheets for Treatment of Osteoporosis

**Figure 6:**
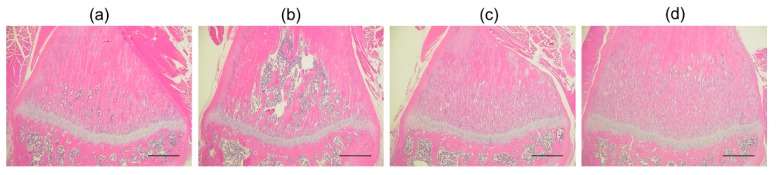
Representative histological micrographs of bone tissue from rat tibias in (**a**) naïve rats, and rats following application of (**b**) ovariectomies (OVX), (**c**) OVX + ALN-loaded HA gel sheets, and (**d**) OVX + subcutaneous administration of ALN. Scale bars represent 500 µm.

Figure 6 shows representative histological sections of bone tissue from the right tibia of OVX rats after treatment with ALN. Compared to that of the naïve rat (Figure 6a), the bone matrix (shown in pink) was decreased 8 weeks after performing OVX (Figure 6b). These results indicate that osteoporosis could be induced in this model. The decrease in bone matrix density was effectively prevented by the subcutaneous injection of ALN in the OVX rats (Figure 6d). Similarly, administration of ALN-loaded HA gel sheets effectively suppressed the decrease in bone matrix density (Figure 6c).

## 4. Discussion

We successfully developed an HA gel sheet that exists as a gel despite containing no gel-forming component other than HA. Although it has been reported that HA gels can be prepared by click chemistry, enzymatic crosslinking, disulfide crosslinking, radical polymerization, condensation reactions, and functionalization of HA with hydrophobic molecules [15,16,17,18,19,20], reports of HA gels obtained by noncovalent interactions of unmodified HA are scarce. In the present study, viscosity measurements using a rotational viscometer indicate that phosphoric acid plays a key role in the HA gelation in our formulation, because the viscosity of HA increased after addition of phosphoric acid. The reversibility of the HA gel sheet–high viscosity state indicates that HA gelation with phosphoric acid is caused by noncovalent interactions. Therefore, we believe that phosphoric acid alters the ratio of the ionic and neutral forms of the functional groups in HA, thus altering the noncovalent interactions (H-bonding, hydrophobic forces, etc.) between these functional groups, which contribute to HA gelation [27], although the detailed gelation mechanism of HA is still not clear. These results, together with the results of the skin irritation study, indicate that our gelation system using phosphoric acid maintains the structure and properties of HA (both its function and biocompatibility). Therefore, when compared with existing chemically-modified, cross-linked HA gel sheets, our HA gel sheet has some advantages, being nonirritating to the skin and easy to prepare.

In the skin permeation and pharmacokinetics studies, we found that efficient skin permeation of ALN was obtained with our HA gel sheet. The steady state of plasma concentration of ALN over 360 min after application of ALN-loaded gel sheet to the rats could be explained by sustainable permeation through the rat skin in vitro. Rat skin is most structurally similar to human skin, and it is the most frequently used rodent model [28]. However, rat skin is generally more permeable than human skin [29,30,31]. Therefore, the human skin drug permeation should also be examined for clinical applications. If the area of the sheet for clinical use is calculated based on the amount of ALN permeating through human skin, the area of the HA gel sheet should be approximately 44–88 cm^2^ to obtain permeation of 315–630 μg ALN over 24 h, which is almost equivalent to the absorbed amount of ALN after oral administration of a 35-mg tablet in clinical use [32]. Because the patches of 50–400 cm^2^, such as the ketoprofen patch, are usually applied to patients in clinical use [33], our ALN-loaded HA gel sheet would be a promising transdermal formulation for clinical applications. In general, drugs with high polarity and hydrophilicity, such as ALN, barely permeate through the skin due to the presence of the stratum corneum as the outer layer. In our previous study, we reported that a hydrophilic patch system with a high concentration of ALN showed efficient skin permeation in a dose-dependent manner [9]. Therefore, the efficient skin permeation of ALN using our HA gel sheet was probably due to the fact that concentrated ALN on the surface of the HA gel sheet generated a high concentration gradient of ALN across the skin, which is critical for driving passive diffusion.

The effective pharmacological effect of ALN was proportional to its efficient skin permeation after application of the ALN-loaded HA gel sheet. Furthermore, the effect of the ALN-loaded HA gel sheet on plasma calcium levels and bone mass was in good agreement with previously reported results from oral and intrapulmonary administration [34]. It was reported that ALN inhibits osteoclast functions, which leads to decreased plasma calcium levels and increased bone mass [35,36,37]. Therefore, our pharmacological study of the ALN-loaded HA gel sheet indicates that pharmacologically active ALN absorbed from the skin is effectively delivered to the bone, where it attenuates bone destruction via osteoclast inhibition.

## 5. Conclusions

In conclusion, we successfully developed a phosphoric acid-mediated HA gel sheet for the efficient transdermal delivery of ALN. We found that phosphoric acid enhanced the noncovalent interactions of HA molecules during HA gelation. The HA gel sheet allowed sufficient skin permeability of ALN for the treatment of osteoporosis. Although the long-term stability of ALN-loaded HA gel sheet and the phosphoric acid-mediated gelation mechanism of HA need to be addressed before clinical use, these results indicate that the phosphoric acid-mediated HA gel sheet is a promising transdermal formulation for the delivery of ALN and the treatment of osteoporosis.

## Figures and Tables

**Table 1:** Effect of phosphoric acid on viscosity of HA–polyhydric alcohol solution.

	Viscosity (Pa·s)	
	Before heated drying	After heated drying
Phosphoric acid (+)	7.90 ± 0.93	out of range
Phosphoric acid (−)	1.45 ± 0.06	50.7 ± 2.38

Results are expressed as the mean ± standard error (SE) of three experiments.

**Table 2:** Pharmacokinetic parameters of ALN.

	Dose (mg/kg)	T_max_ (min)	C_max_ (ng/mL)	AUC (μg·min/mL)	BA (%)
Intravenous injection	1	-	-	105 ± 12	-
ALN-loaded HA gel sheet	2.5	720 ± 360	53 ± 10	50 ± 6.5	20 ± 2.6

T_max_, time to maximum plasma concentration; C_max_, maximum plasma concentration; AUC, area under the concentration–time curve; BA, bioavailability. Results are expressed as the mean ± standard error (SE) of three experiments.
